# Using proteomic profiling to characterize protein signatures of different thymoma subtypes

**DOI:** 10.1186/s12885-019-6023-4

**Published:** 2019-08-13

**Authors:** Liang-Chuan Lai, Qiang-Ling Sun, Yu-An Chen, Yi-Wen Hsiao, Tzu-Pin Lu, Mong-Hsun Tsai, Lei Zhu, Eric Y. Chuang, Wentao Fang

**Affiliations:** 1 Graduate Institute of Physiology, College of Medicine, National Taiwan University, Taipei, 10051 Taiwan; 2 Department of Thoracic Surgery, Shanghai Chest Hospital, Shanghai Jiao Tong University, Shanghai, 200030 China; 3 Bioinformatics and Biostatistics Core, Center of Genomic and Precision Medicine, National Taiwan University, Taipei, 10055 Taiwan; 4 Department of Public Health, National Taiwan University, Taipei, 10055 Taiwan; 5 Institute of Biotechnology, National Taiwan University, Taipei, 10672 Taiwan; 6 Graduate Institute of Biomedical Electronics and Bioinformatics, National Taiwan University, Taipei, 10617 Taiwan; 7 Department of Pathology, Shanghai Chest Hospital, Shanghai Jiao Tong University, Shanghai, 200030 China; 8 Department of Electrical Engineering, Graduate Institute of Biomedical Electronics and Bioinformatics, National Taiwan University, Taipei, 106 Taiwan

**Keywords:** Proteomic profiling, Thymoma, Support vector machine, WHO classification

## Abstract

**Background:**

Histology is a traditional way to classify subtypes of thymoma, because of low cost and convenience. Yet, due to the diverse morphology of thymoma, this method increases the complexity of histopathologic classification, and requires experienced experts to perform correct diagnosis. Therefore, in this study, we developed an alternative method by identifying protein biomarkers in order to assist clinical practitioners to make right classification of thymoma subtypes.

**Methods:**

In total, 204 differentially expressed proteins in three subtypes of thymoma, AB, B2, and B3, were identified using mass spectrometry. Pathway analysis showed that the differentially expressed proteins in the three subtypes were involved in activation-related, signaling transduction-related and complement system-related pathways. To predict the subtypes of thymoma using the identified protein signatures, a support vector machine algorithm was used. Leave-one-out cross validation methods and receiver operating characteristic analysis were used to evaluate the predictive performance.

**Results:**

The mean accuracy rates were > 80% and areas under the curve were ≧0.93 across these three subtypes. Especially, subtype B3 had the highest accuracy rate (96%) and subtype AB had the greatest area under the curve (0.99). One of the differentially expressed proteins COL17A2 was further validated using immunohistochemistry.

**Conclusions:**

In summary, we identified specific protein signatures for accurately classifying subtypes of thymoma, which could facilitate accurate diagnosis of thymoma patients.

## Background

Epithelial tumors of the thymus include thymoma and thymic carcinoma. Compared to thymic carcinoma, thymoma tends to recur locally and is frequently associated with autoimmune diseases, such as myasthenia gravis. The incidence rate of thymoma is around 2.5 per million people per year [1]. The age distribution ranges from 10 to 80 years old with an average age of 50 to 60 in both males and females [1].

In the quest for accurate diagnosis, treatment, and prognosis for thymoma patients, researchers use clinical data to classify thymoma into different types. In 1999, the World Health Organization (WHO) proposed a classification system that divided thymic tumors into six types according to the morphology of epithelial cells and the lymphocyte-to epithelial cell ratio: thymoma type A, AB, B1, B2, and type C [2]. Type C thymoma was then revised into thymic carcinoma instead in the 2004 version of WHO thymoma classification standard [3, 4].

In this system, the existence of spindled or epithelioid neoplastic epithelial cells and the amount of lymphocytes was used as a basic classifying criterion. Also, Suster et al. proposed a thymoma classification system based on the differentiation level of tumor cells [5]. In this classification system, thymoma, atypical thymoma, and thymic carcinoma are defined as highly, moderately, and poorly differentiated, respectively. However, distinguishing thymic tumors merely through histological observations is still quite complicated because of the diverse morphology of thymoma and the presence of non-neoplastic lymphocytes. For example, type AB, B2 and B3 are all admixed with various amount of lymphocytes, but the prognosis of type AB was different from those of type B2 and B3 [6]. Furthermore, due to the rarity of the disease, lack of experience among pathologists in general were quite common. Therefore, Suster et al. has reviewed several classification systems of thymic epithelial neoplasms, including Suster & Moran, WHO, Kirchner & Muller-Hermelink, and Bernatz, and suggested that the future of thymoma classification should include a simplified nomenclature rather than increasing complexity of histopathologic classification [2, 5, 7–9].

With the development of sequencing techniques in the twenty-first century, the Cancer Genome Atlas (TCGA) also provide thymoma sequencing data for genomic exploration [10, 11]; and the Clinical Lung Cancer Genome Project [12] as a genetic foundation for more accurate classification [13]. Also, Zettl et al. performed comparative genomic hybridization in 28 thymoma and 9 thymic squamous cell carcinoma cases. They found distinct genetic phenotypes between thymoma type A and type B3 [14]. Lee et al. also performed comparative genomic hybridization in 39 thymoma cases, and identified a set of 33 genes that could be divided into 4 genetically distinct groups (A, AB, B1 + 2, and B3) according to the WHO classification, where type AB was determined to be genetically heterogeneous [15]. These reports indicate the importance of and trend toward integrating clinical data with genomics and proteomics data for a more comprehensive understanding of thymoma classification.

Therefore, the purpose of this study was to classify three thymoma types (AB, B2, and B3), which were difficult to classify histologically, by analyzing the proteomics data of 21 Chinese patients. In total, 204 differentially expressed proteins were detected in the three subtypes of thymoma, and their functions were identified. Also, a predictive model for thymoma subtype was created, and leave-one-out cross validation (LOOCV) methods and receiver operating characteristic (ROC) curve analysis were used to evaluate the performance of the predictive model. Lastly, immunohistochemistry was used for validation.

## Methods

### Collection and processing of clinical material and patient information

Fresh tumor and adjacent normal tissue specimens were obtained during surgery between 1994 and 2010 and snap-frozen. The specimens were obtained from 21 patients treated at the Shanghai Chest Hospital, Shanghai Jiao Tong University, Shanghai, China. Tumor histology was determined according to the WHO classification [16] by an experienced pathologist (LZ) who reviews over a hundred cases of thymic malignancies annually. Information regarding age, gender, pathological stage, and WHO classification was collected prospectively. The study was approved by the Institutional Review Board of Shanghai Chest Hospital. And written consent was acquired from all patients included in this study.

### Mass spectrometry analysis

In contrast to shotgun mass spectrometry (MS) and traditional database searches, the quantitative proteomic profiling of type AB, type B2 and type B3 tissues were performed by using Data Independent Acquisition Mass Spectrometry (DIA-MS) to expand the detectable dynamic range and improve the overall confidence of protein quantification measurements. Fresh frozen tissues were lysed and subjected to reduction, alkylation, and tryptic digestion. Peptides were then subjected to desalt using C18 SPE 96-well plates prior to LCMS/MS analysis using Orbitrap Fusion (Thermo Scientific). One μg peptides was loaded onto a nano-C18 column and separated at a flow rate of 300 nL/min. Data independent high-resolution MS/MS spectra were acquired by sequential 25 amu window. Protein identification and quantification were processed by Spectronaut software (Biognosys Inc.). Differential expressed proteins were subjected to bioinformatics analysis.

### Data analysis

Raw protein expression intensities were preprocessed by log_2_ transformation and then normalized by quantile normalization. To remove the background noise, proteins whose normalized intensity was below 32 were removed from further analysis, which accounted for ~ 2.5% of the total protein in each sample. Then, differentially expressed proteins (DEPs) between tumor and adjacent normal tissue in each subtype were identified by log_2_ fold-change value ≧7 and *P*-value < 0.05 using Wilcoxon signed-rank test. After selecting DEPs from each subtype, the total and unique (subtype-specific) DEPs were identified and visualized by Venn diagrams. Principal component analysis using the expression values of total DEPs was used to visualize the similarity of different samples. An unsupervised hierarchical clustering method was applied using Euclidean distance and the method of average linkage in Genesis version 1.8.1 (http://genome.tugraz.at/genesisclient/genesisclient_description.shtml). Ingenuity Pathway Analysis (IPA) was used to identify enriched pathways of DEPs for each subtype.

To predict the subtypes of thymoma by identified protein signatures, a support vector machine (SVM) algorithm, a supervised learning model, was used. The unique DEPs in each subtype were chosen as a set of features for training the classifiers. In this study, our classifiers are the three thymoma subtypes, the closer to real results in the prediction, the more accurate classifiers they are. During the LOOVC analysis, each set of unique DEPs of one subtype took turns serving as testing data, whereas the remaining two sets served as training data. After the predictive models were established (three models in total), all three subtypes were then served as three sets of sample data, yielding nine predictive results. The robustness of each classifier was estimated by the average classification accuracy rate in each validation. After LOOCV, the sensitivity, specificity, positive predictive value, and negative predictive value of the prediction models for each subtype were calculated. The evaluation of these parameters was performed using ROC curve analysis, the result of which was quantified by computing the area under the curve (AUC).

### Immunohistochemical analysis

Five type AB and five type B2/B3 thymoma formalin-fixed and paraffin-embedded (FFPE) tissue sections were used to examine the presence of COL17A2. Briefly, slides were deparaffinized with diaminobenzene at 65 °C for 2 h, and rehydrated in graded alcohol solutions. The tissue sections were then treated with 3% hydrogen peroxidase to block endogenous peroxidase activity. After washing in PBS solution, the tissue sections were incubated in 20% normal goat serum at 37 °C for 10 min. Rabbit monoclonal antihuman COL17A2 antibody (Abcam ab184996) was applied at 1:100 dilution overnight. Biotin- and streptavidin-labeled antibodies were used for 3,3′-diaminobenzidine staining. Nucleus was further stained with hematoxylin. Images were taken by ZEISS fluorescent microscope.

The score system was based on the immunoreactive intensity to COL17A2 antibody and the percentage of positively stained cells. The stained intensity was scored from a value from 0 to 3. The percentage of positively stained cells were the average score in 5 fields (100 cells per field in 400x magnitude). Score 4 was given for > 75% of positive stained cells; score 3 for 75–51% of cells positive; score 2 for 50–26% of cells positive; score 1 for 25–6% of cells positive; score 0 when less, than 5% of tumor cells or no visible staining was observed. The immunoreactive score is determined by multiplication of the score of staining intensity with the score of percentage of positively stained cells.

## Results

### Patients

The clinicopathological characteristics of the 21 thymoma patients included in this study are summarized in Table 1. The median of age was 51. Tumor stages and histology subtypes were defined by Masaoka-Koga system [17] and WHO classification separately. There was no significant difference in the distribution of patients regarding gender, stage, or subtype according to WHO classification.

**Table 1:** Clinicopathological characteristics of patients with thymoma. Tumor stages are defined by Masaoka-Koga classification

Factors	Number (%)
Age	
≧60	6 (28.6)
< 60	15 (71.4)
Gender	
Male	10 (47.6)
Female	11 (52.4)
Stage	
I	9 (42.9)
II	7 (33.3)
III	2 (9.5)
IV	3 (14.3)
WHO classification	
AB	8 (38.1)
B2	8 (38.1)
B3	5 (23.8)

### Identification of differentially expressed proteins

**Fig. 1:**
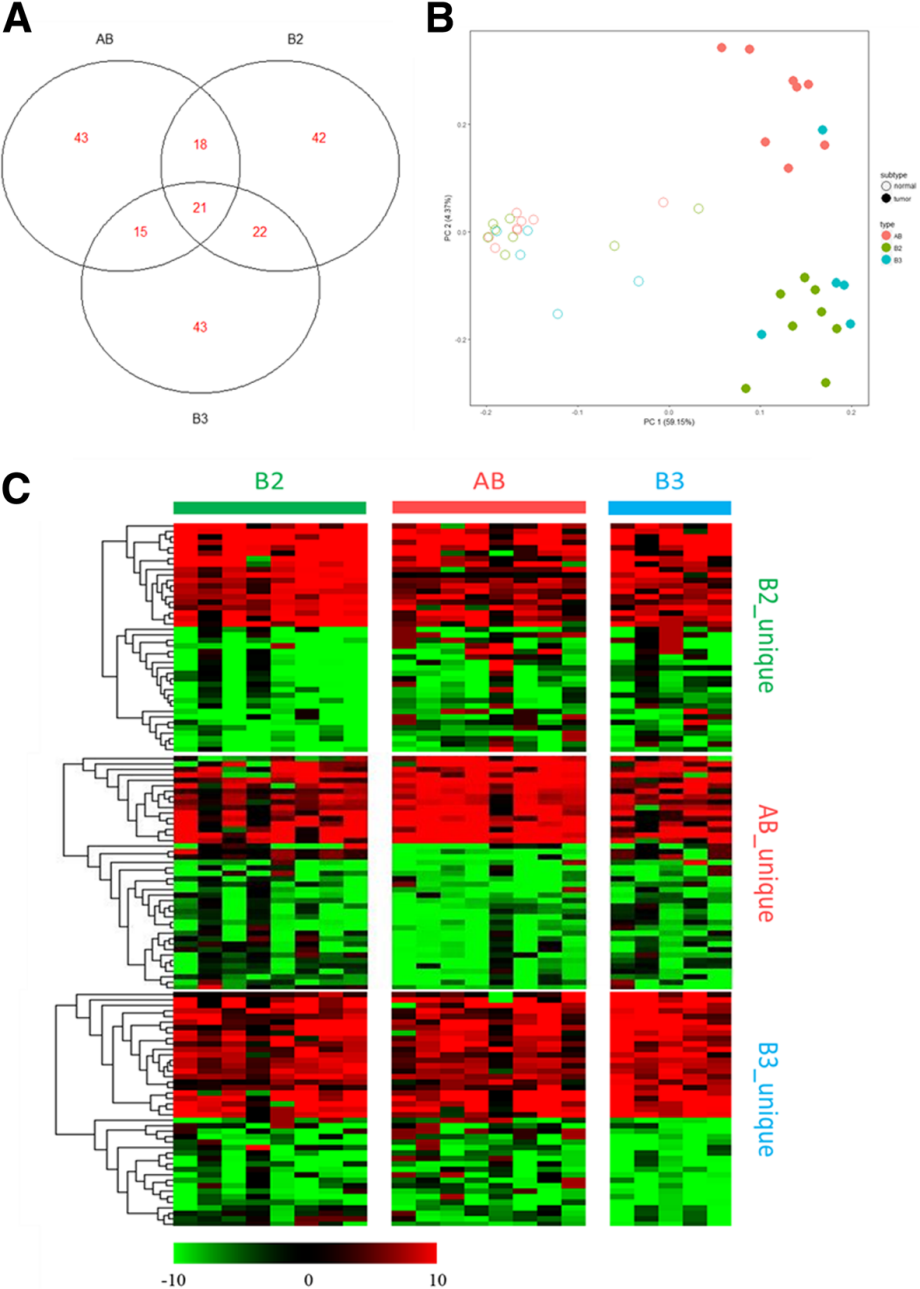
Analysis of differentially expressed proteins in different types of thymomas. **a** Venn diagram of differentially expressed proteins (DEPs) among 3 types of thymoma. **b** Principal component analysis of 21 samples using the complete set of DEPs (n = 204). Solid and hollow circles respectively stand for tumor and normal samples, and AB (pink), B2 (green), and B3 (cyan) subtypes were presented in different colors. **c** Expression profiling of DEPs. Red: up-regulation in tumor tissues as compared to its adjacent normal tissues; Green: down-regulation

In total, 204 DEPs between tumor and adjacent normal tissue of the 3 thymoma subtypes were identified through statistical filtering (Table 2). The number of DEPs detected in AB, B2, and B3 subtypes was 97, 103, and 114, respectively. The number of up−/down-regulated DEPs and unique (subtype-specific) DEPs is shown in Table 2. The distribution of the total number of DEPs among shared and unique DEPs is shown in Fig. 1a.

**Table 2:** Number of differentially expressed proteins between thymoma and adjacent normal tissue

	Thymoma Type		
	AB	B2	B3
All proteins	3118		
DEP (T vs N)^*a,b*^	97	103	114
Up-regulation	40	33	34
Down-regulation	57	70	80
Unique	43	42	37

^*a*^ T: tumor tissue; N: adjacent normal tissue

^*b*^ DEP: differentially expressed protein. Selection criteria: absolute log_2_ fold-change value ≥7 and a *P*-value ≤0.05 using a Wilcoxon signed-rank test

### Cluster analysis

Principal component analysis of the 21 paired samples (tumor versus normal, 42 dots in total) was performed using a total of 204 DEPs in 3 subtypes (Fig. 1b). The normal and tumor samples were clearly divided into 2 main groups, and within the tumor cluster, the B2 and B3 subtypes were further distinguished from the AB subtype, with the exception of one B3 sample appearing with the AB cluster. This result suggests a distinct protein expression pattern in the AB subtype of thymoma as compared to the other 2 subtypes.

Hierarchical cluster analysis was performed within each subtype-unique DEPs set (Fig. 1c). The expression level of each unique DEPs set (labeled on the right) in all three subtypes (labeled on the top) was presented by heatmap. The expression difference of each unique DEPs set was apparently greater in their own subtype than in other 2 subtypes, confirming the existence of unique sets of DEPs for each subtype.

### Pathway analysis

IPA revealed the biological pathways in which the DEPs participated (Table 3). A –log *P*-value of Fisher’s exact test and the ratio of DEPs to the total number of proteins in the same pathway were used to estimate the significance and importance of each pathway. Among the top 5 canonical pathways in each thymoma subtype (15 pathways total), the most frequently occurring were signal transduction (6 pathways), LXR/RXR or FXR/RXR activation (4 pathways), and complement system (2 pathways).

**Table 3:** Pathway analysis of differentially expressed proteins for each subtype of thymoma

Canonical Pathway^*a*^	-log(*P*-value) ^*b*^	Ratio^*c*^
*Subtype AB*		
Acute Phase Response Signaling	6.96	0.05
Complement System	3.18	0.08
Apoptosis Signaling	3.07	0.04
Triacylglycerol Degradation	3.05	0.08
Granzyme B Signaling	2.59	0.13
*Subtype B2*		
LXR/RXR Activation	8.06	0.07
FXR/RXR Activation	6.71	0.06
Atherosclerosis Signaling	5.57	0.06
Production of Nitric Oxide and Reactive Oxygen Species in Macrophages	4.31	0.04
IL-12 Signaling and Production in Macrophages	4.10	0.04
*Subtype B3*		
Acute Phase Response Signaling	12.00	0.08
Complement System	7.83	0.17
Coagulation System	4.69	0.11
LXR/RXR Activation	4.68	0.05
FXR/RXR Activation	4.60	0.05

^*a*^ Canonical pathway analysis was conducted by the Ingenuity® Pathway Analysis (IPA) program and analyzed based on the Ingenuity® Knowledge Base (Content version: 39480507; Release date: 2017-09-14)

^*b*^ Fisher’s exact test was used to determine the enrichment of differentially expressed proteins in a given canonical pathway

^*c*^ Ratio represents the number of differentially expressed proteins in the pathway divided by the total number of proteins in the same pathway

When comparing common pathways between subtypes, subtypes B2 and B3 had 2 common pathways: LXR/RXR activation (−log *P-value =* 8.06 (B2) and 4.68 (B3)) and FXR/RXR activation (−log *P*-value = 6.71 (B2) and 4.60 (B3)). Subtypes AB and B3 also had 2 common pathways: acute phase response signaling (−log *P*-value = 6.96 (AB) and 12.00 (B3)) and complement system (−log *P*-value = 3.18 (AB) and 7.83 (B3)).

Subtype AB had the most similar pathways, where 3 of the top 5 canonical pathways were signaling-related: acute phase response signaling, apoptosis signaling, and granzyme B signaling (−log *P*-value = 6.96, 3.07 and 2.59).

### Predicting thymoma subtypes with unique DEPs signatures

**Fig. 2:**
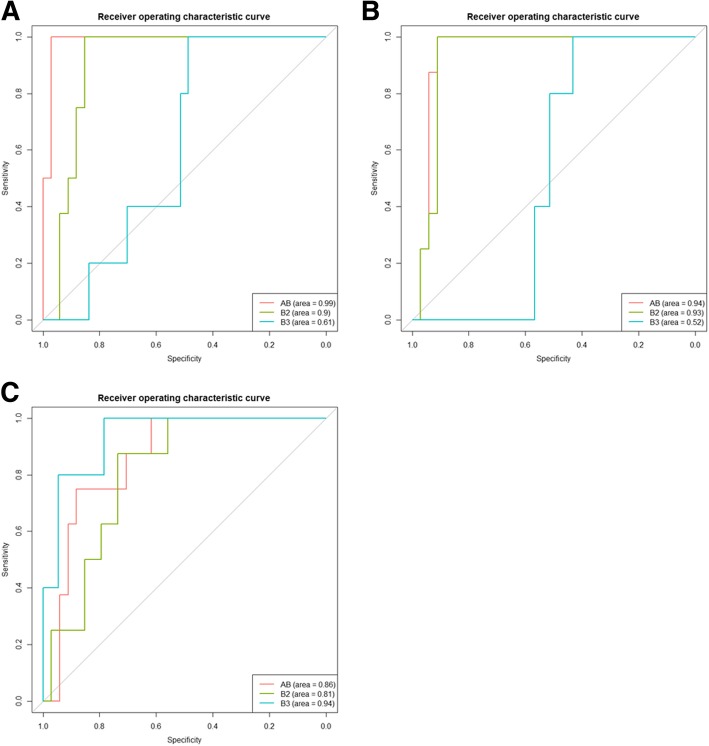
Receiver operating characteristic curve of SVM classifiers to predict the type of thymoma. Differentially expressed proteins only in types AB (**a**), B2 (**b**), and B3 (**c**) were served as test data to train different classifiers

The results of thymoma subtype prediction by a supervised learning model with existing unique protein signatures are shown in Table 4. The accuracy rate of LOOCV for all 3 subtypes was > 80%, where B3 had the highest accuracy rate (96%). Sensitivity, specificity, positive predictive value and negative predictive value are also shown (Table 4). The prediction efficiency evaluations of the diagnostic test by ROC analysis are shown in Fig. 2, with the AUCs of the SVM classifiers in Table 4. Overall, AB (AUC = 0.99) seemed to be the best classifier among the 3 subtypes when comparing to other two subtypes, B2 (AUC = 0.93) and B3 in Fig. 2.

**Table 4:** Summary of thymoma subtype prediction by a LOOCV method

	Thymoma Type		
	AB	B2	B3
No. of Samples	8	8	5
No. of Unique DEP^*a*^	43	42	37
LOOCV mean accuracy rate^*b*^	83%	83%	96%
Sensitivity	0.89	0.73	0.50
Specificity	1.00	1.00	0.86
PPV^*c*^	100%	100%	38%
NPV^*d*^	97%	91%	91%
AUC^*e*^	0.99	0.93	0.94

^*a*^ DEP: differentially expressed protein

^*b*^The average classification accuracy rate was calculated in leave-one-out cross validation using differentially expressed proteins

^*c*^ PPV: positive predictive value, the proportion of positive results in a classifier that is truly positive in the experimental results

^*d*^ NPV: negative predictive value, the proportion of negative results in a classifier that is truly negative in the experimental results

^*e*^ AUC: Area under the curve, represents how well a classifier can distinguish one type from the others

**Fig. 3:**
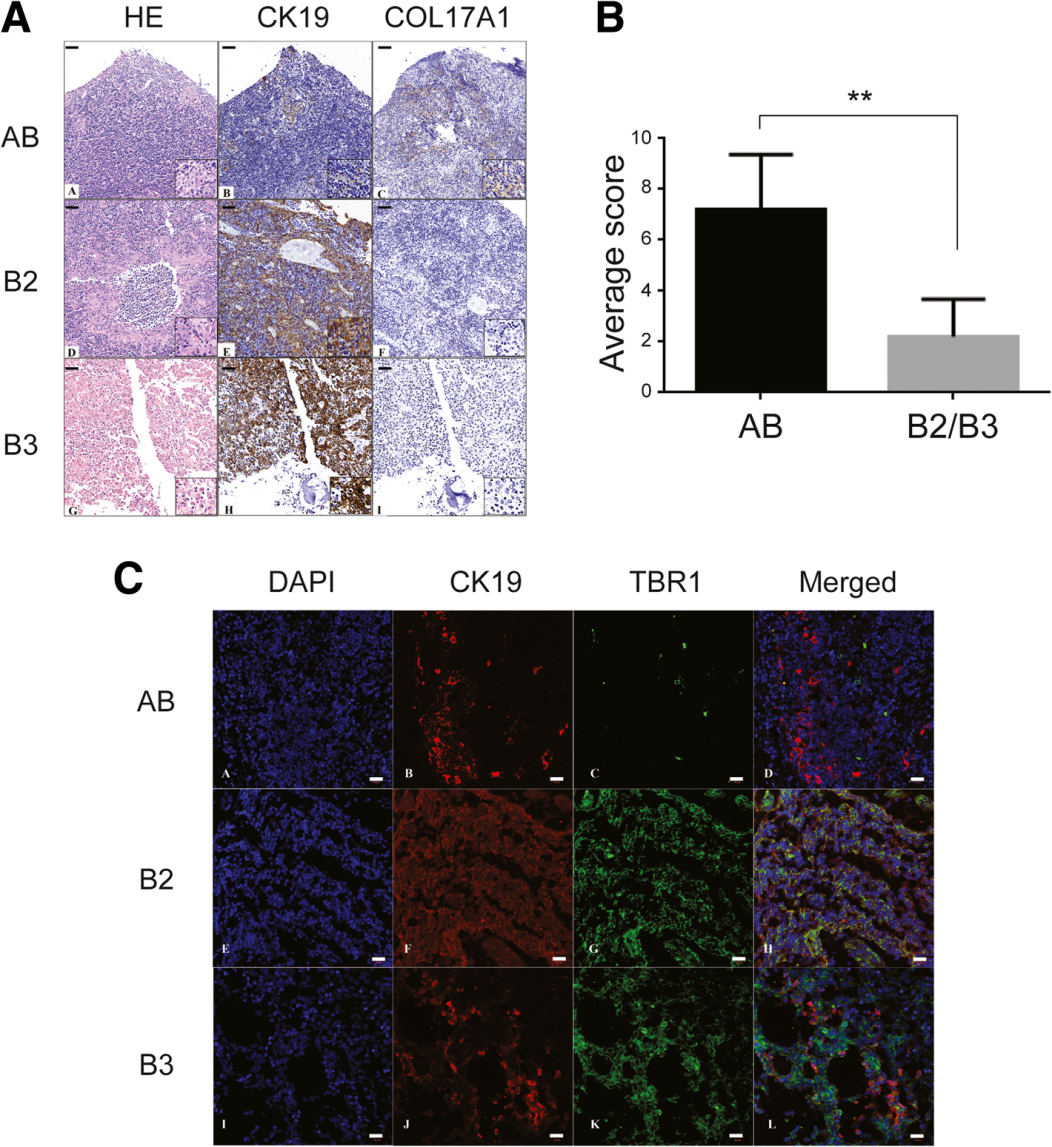
Immunochemical analysis of COL17A1 and TBR1 in type AB and B2/B3 of thymoma. (**a**) immunohistochemistry of COL17A1. Formalin-fixed and paraffin-embedded (FFPE) tissue sections were stained to examine the presence of COL17A1. Scale bar: 100 μm. Insert magnification: 400X (**b**) Quantification of COL17A1. The immunoreactive score is determined by multiplication of the score of staining intensity with the score of percentage of positively stained cells. **, *P* < 0.01. **c** Immunofluorescence of TBR1. CK19 (Cytokeratin 19): epithelial marker of thymoma. Scale bar: 20 μm

Lastly, we selected two of differentially expressed proteins COL17A1 (Collagen Type XVII Alpha 1 Chain) and TBR1 (T-Box, Brain 1) for validation using immunochemical analysis. Based on the results of mass spectrometry, COL17A1 was only up-regulated in type AB, but not in type B2/B3, and had the greatest fold-change (~920X). As shown in Fig. 3, COL17A1 was differentially expressed in subtype AB using immunohistochemistry (Fig. 3a). The immunoreactive score is determined by multiplication of the score of staining intensity with the score of percentage of positively stained cells. The amount and the percentage of thymoma containing COL17A1 was significantly (*P* < 0.01) higher in type AB as compared to type B2/B3 (Fig. 3b). In addition, immunofluorescence of TBR1 validated the results of mass spectrometry (Fig. 3c), which showed TBR1 had significantly higher expression in type B2/B3 than type AB.

## Discussion

Because of the diverse morphology of thymoma and the rarity of the disease, classification of thymoma solely based on histology is challenging. Here we used proteomics information to identify DEPs in different thymoma subtypes in order to classify them. This method can be used to customize the diagnosis, treatment, and prognosis of specific thymoma subtypes. In our results, distinct protein expression patterns of thymoma subtype AB were revealed, as compared to B2 and B3 subtypes, in both cluster analysis and pathway analysis. Furthermore, subtype AB was also the best classifier among the 3 subtypes based on the ROC analysis.

In the beginning of the analysis, we excluded partial proteins (< 5% of total protein) as background noise. Although this may have slightly affected the results of the pathway analysis, small changes in these partial proteins often lead to tremendous fold changes and result in false positives. Moreover, although subtype AB, B2 and B3 are all mixed with various amount of lymphocytes, the contents of lymphocytes are not a concern because the classification is based on the differentially expressed proteins in each subtype. If the differentially expressed proteins come from lymphocytes, they are part of characteristics for classifying the subtypes of thymoma. Thirdly, although protein expression levels could change at different tumor stages, we did not consider stage as a factor in our analysis. The main reason for this was that the samples used for compiling the data set showed no significant deviation from randomness in the distribution across stages, and the main focus of this study was to classify subtypes of thymoma.

According to the “hallmarks of cancer” proposed by Hanahan and Weinberg in 2000, the molecular abnormalities of malignancies can be summed up in 6 characteristics: self-sufficiency in growth signaling; insensitivity to antigrowth signals; evading apoptosis; limitless replicative potential; sustained angiogenesis; and tissue invasion and metastasis [18]. In our study, pathway analysis revealed several canonical pathways related to signal transduction (6 of 15 pathways), nuclear receptor activation (4 of 15 pathways), and complement system (2 of 15 pathways) in the 3 thymoma types, which correlate with Hanahan and Weinberg’s hallmarks of cancer.

The similarity of pathways between subtypes could be caused by similar genetic expression profiles between samples. As mentioned earlier, LXR/RXR activation and FXR/RXR activation were observed in common in subtypes B2 and B3, whereas acute phase response signaling and complement system were in common in subtypes AB and B3. These results correlate with the clustering analysis, where AB was separate from the B2 + B3 cluster, but closer to B3 than to B2.

The similarity between the top canonical pathways in subtype AB—acute phase response signaling, apoptosis signaling, and granzyme B signaling (−log *P*-value = 6.96, 3.07 and 2.59)—suggests that the genetic expression profile changes in subtype AB are highly related to signal transduction.

ROC analysis is useful for assessing the utility of predictors in clinical metrics and diagnostics tests. It is widely used in epidemiology and diagnostic radiology research [19–21]. So far this method has not yet been performed for thymic tumor classification. As far as we know, this is the first study applying ROC analysis to classify different types of thymoma. In our study, the AUC in all 3 tumor types was ≥0.93, implying high performance of these classifiers. Despites of its high performance and being scrutinized by leave-one-out cross validation, if there are any other independent cohort available, it will still be a good reason to further improve its reliability without the use of original samples as statistical assessment. However, because a rare disease thymoma is, the difficulty of doing so is also higher than other common diseases.

In this study, we analyzed the protein mass spectrometry data of 21 Chinese patients with 3 types of thymoma (AB, B2 and B3), and developed a thymoma subtype predictive model with overall AUC ≥0.93. Although there probably does not exist any geographic or racial difference in terms of histological classification of thymomas, the use of single human race may still be a limitation in this study.

We expect to combine more clinical data (e.g., survival data) in the future to improve the predictive model. Since the current dilemma in classifying types of thymoma is the diverse clinical features, generation of a predictive model with good performance will be a considerable contribution to this field of study.

## Conclusions

In this study, we identified protein signatures to predict the subtypes AB, B2/B3 of thymoma with high accuracy, sensitivity and specificity. One of the differentially expressed proteins COL17A1 was further validated using immunohistochemistry. These specific protein signatures could facilitate accurate diagnosis of thymoma patients.

## Data Availability

All data generated or analyzed during this study are included in this published article.

## Abbreviations

DEP: Differentially expressed protein
FFPE: Formalin-fixed and paraffin-embedded
IPA: Ingenuity Pathway Analysis
LOOCV: Leave-one-out cross validation
ROC: Receiver operating characteristic
TCGA: The Cancer Genome Atlas
WHO: World Health Organization
